# Impact of treatment planning and delivery factors on gastrointestinal toxicity: an analysis of data from the RADAR prostate radiotherapy trial

**DOI:** 10.1186/s13014-014-0282-7

**Published:** 2014-12-13

**Authors:** Noorazrul Yahya, Martin A Ebert, Max Bulsara, Annette Haworth, Rachel Kearvell, Kerwyn Foo, Angel Kennedy, Sharon Richardson, Michele Krawiec, David J Joseph, Jim W Denham

**Affiliations:** School of Physics, University of Western Australia, Crawley, Western Australia Australia; School of Health Sciences, National University of Malaysia, Bangi, Malaysia; Department of Radiation Oncology, Sir Charles Gairdner Hospital, Nedlands, Western Australia Australia; Institute for Health Research, University of Notre Dame, Fremantle, Western Australia Australia; Department of Physical Sciences, Peter MacCallum Cancer Centre, Victoria, Australia; Sir Peter MacCallum Department of Oncology, University of Melbourne, Victoria, Australia; Sydney Medical School, University of Sydney, Sydney, New South Wales Australia; School of Surgery, University of Western Australia, Crawley, Western Australia Australia; School of Medicine and Public Health, University of Newcastle, Newcastle, New South Wales Australia

**Keywords:** Gastrointestinal toxicity, Prostate cancer, Technical modifications, Dose-volume histogram

## Abstract

**Background:**

To assess the impact of incremental modifications of treatment planning and delivery technique, as well as patient anatomical factors, on late gastrointestinal toxicity using data from the TROG 03.04 RADAR prostate radiotherapy trial.

**Methods:**

The RADAR trial accrued 813 external beam radiotherapy participants during 2003–2008 from 23 centres. Following review and archive to a query-able database, digital treatment plans and data describing treatment technique for 754 patients were available for analysis. Treatment demographics, together with anatomical features, were assessed using uni- and multivariate regression models against late gastrointestinal toxicity at 18-, 36- and 54-month follow-up. Regression analyses were reviewed in the context of dose-volume data for the rectum and anal canal.

**Results:**

A multivariate analysis at 36-month follow-up shows that patients planned using a more rigorous dose calculation algorithm (DCA) was associated with a lower risk of stool frequency (OR: 0.435, CI: 0.242–0.783, corrected *p* = 0.04). Patients using laxative as a method of bowel preparation had higher risk of having increased stool frequency compared to patients with no dietary intervention (OR: 3.639, CI: 1.502–8.818, corrected *p* = 0.04). Despite higher risks of toxicities, the anorectum, anal canal and rectum dose-volume histograms (DVH) indicate patients using laxative had unremarkably different planned dose distributions. Patients planned with a more rigorous DCA had lower median DVH values between EQD2_3_ = 15 Gy and EQD2_3_ = 35 Gy. Planning target volume (PTV), conformity index, rectal width and prescription dose were not significant when adjusted for false discovery rate. Number of beams, beam energy, treatment beam definition, positioning orientation, rectum-PTV separation, rectal length and mean cross sectional area did not affect the risk of toxicities.

**Conclusions:**

The RADAR study dataset has allowed an assessment of technical modifications on gastrointestinal toxicity. A number of interesting associations were subsequently found and some factors, previously hypothesised to influence toxicity, did not demonstrate any significant impact. We recommend trial registries be encouraged to record technical modifications introduced during the trial in order for more powerful evidence to be gathered regarding the impact of the interventions.

**Electronic supplementary material:**

The online version of this article (doi:10.1186/s13014-014-0282-7) contains supplementary material, which is available to authorized users.

## Introduction

Technical modifications in radiotherapy are often incremental, introduced into practice in institutions as evidence emerges and technological capability materializes. Planning studies are used widely to quantify the superiority of the modifications on the basis of planned dose distributions [1]. Such studies assume a relationship between dose precision and what is considered a ‘better’ dose distribution and the actual impact the modifications have on patients.

For example, more rigorous dose calculation algorithms (DCA) are introduced because of their ability to correct for tissue heterogeneity [2] while more sophisticated beam modification devices allow for increased dose conformality around the target volume, resulting in a lower dose to surrounding healthy tissue. Although these treatment modifications are introduced into clinical practice after careful consideration of their dosimetric consequences, ambiguity can persist regarding their clinically relevant consequences.

Due to the incremental and uncritical nature of technical changes, their clinical impacts are only occasionally assessed by way of hypothesis via randomised clinical study of the factors themselves [3-5]. More frequently, treatment-related morbidities have been examined via theoretical analysis using data collected during a study for which such factors comprise covariates [6-9]. These studies were either single institutional [3,4,6,7] or accruing from a small number of institutions [8]. Few, if any, have reported the benefits of these incremental advances in technique in the multicentre, randomised clinical trial setting.

Randomised controlled trials are typically not powered to determine the effect of these technical modifications on toxicity. Secondary analysis does however offer an alternative way to acquire evidence of probable clinical consequences [10], taking full advantage of the availability of such data to understand how treatment decisions affect outcomes [11]. Long follow-up time associated with randomised clinical trials may be able to close the clinical evidence gap introduced by incremental evolution in radiotherapy, known to be rather rapid. Furthermore, through collaborative efforts, meta-analyses of similar high quality clinical trial data has the potential to provide additional evidence to support the gain in clinical efficacy with the introduction of new technology.

The TROG 03.04 trial of Randomised Androgen Deprivation and RT (RADAR - NCT00193856) principally examined the impact of duration of androgen suppression (AS) therapy on intermediate and high-risk prostate cancer patients [12,13]. All patients had adjuvant RT and, as part of an extensive technical quality assurance (QA) program [14-16], considerable data was collected on treatment technique. With maturity of late toxicity information, these data have now been explored for any significant relationships between treatment planning and delivery factors and resulting gastrointestinal (GI) toxicity.

## Methods and materials

### Data collection

The RADAR trial examined the influence of duration of AS with or without bisphosphonate treatment, adjuvant with radiotherapy. Data collection, protocol requirements and QA have been summarised previously [13,15-17]. This trial commenced accrual at a time when many participating centres were implementing conformal three-dimensional (3D) treatment technologies and was consequently undertaken under detailed scrutiny of patient safety and treatment quality.

Of 1071 men accrued to the trial from 23 participating centres across Australia and New Zealand between 2003 and 2008, 813 had external beam radiotherapy (without a brachytherapy boost) and, of these, 754 had complete technical data available for the analysis presented here, comprising:

- Digital treatment plan export consisting of axial computed tomography (CT) slices at maximum 5 mm spacing; delineated clinical target volume (CTV - the prostate for intermediate-risk patients, and prostate with proximal seminal-vesicles for high-risk), planning target volume (PTV) for phase 1 treatment to 46 Gy (PTV1 – CTV +1.0 - 1.5 cm margin, 0.5 – 1.0 cm posterior margin) and phase 2 boost (PTV2 – CTV +0.0 – 1.0 cm margin, < 0.5 cm posterior margin) to 66, 70 or 74 Gy; delineated outer rectal wall; 3D dose matrix for each phase; information on number of beams, beam energies and collimation method; utilised dose DCA; conformity index (CI) [18]
- Form-based information on patient, centre and treatment demographics, setup technique and dietary interventions.

Factors could be directly extracted, or derived, from the resulting archived data sets using the plan review software, ‘SWAN’ (Sir Charles Gairdner Hospital, Nedlands, WA, Australia) [19]. Several of these require a brief definition or description.

- **Dose calculation algorithm**: these have been categorised as either ‘type-*a*’ or ‘type-*b*’ according to the definitions of Fogliata *et al.* [2]. Briefly, type-*a* algorithms do not account for changes in lateral electron transport due to inhomogeneity whereas type-*b* algorithms do account of such changes.
- **Treatment beam definition:** categorised as either ‘MLC’ (if defined by multileaf collimator), ‘collimator’ (if defined by secondary photon jaws only) or ‘blocks’ (if patient-specific custom-formed blocks were used).
- **Setup orientation**: refers to the orientation (prone or supine) of the patient at the time of planning CT scan, consistent with that at treatment.
- **Rectal dietary intervention**: refers to the intervention in each centre prior to planning CT and each treatment fraction, broadly categorised as ‘no intervention’, ‘laxative prior to planning CT and at least first week of treatment’ and ‘bulking agent with or without dietary instructions’. In all groups, patients were required to empty their rectum before planning and treatment.
- **PTV-rectum separation**: the distance between the anterior extent of the outer rectal wall and the PTV at the axial level of the centre-of-mass of the PTV (which may be negative if the two definitions intersect at that level).
- **95% isodoses volume**: volume, in cm^3^, of the 95% isodose for combined physical phase 1 and 2 isodoses.
- **Conformity index (CI)**: Volume enclosed by 95% isodose surface/PTV [18].
- **Rectal distension**: the normal distance between the most anterior and most posterior coronal planes of the rectum containing points of the outer rectal wall at the level of the centre-of-mass of the gross tumour volume.
- **Mean rectal cross-sectional area (CSA)**: obtained by dividing the total delineated rectum volume by the number of CT slices defining the cranio-caudal length of the rectum (see [20])

### Toxicity assessment

After treatment, all patients were routinely followed up in clinic every 3 months for the first 18 months, then every 6 months up to 5 years post randomisation and then annually for a further 5 years. GI toxicities were assessed using two standard toxicity measurement apparatus; Common Toxicity Criteria v2.0 [21] for proctitis, and LENT-SOMA [22] for stool frequency, tenesmus, and rectal bleeding. Due to the low number of patients reporting high level toxicity, the grade cut-point was adjusted to include patients reporting low level toxicity as shown on Table 1. Patients with pre-treatment symptoms were excluded for that particular endpoint. As the cumulative incidence analysis may overestimate the actual toxicity burden and may have higher level of noise [23,24], the prevalence was used in the current analysis. The toxicity prevalence at 18, 36 and 54 months after randomisation were presumed to be representative of the late toxicity. For the rest of this paper, proctitis (P) at 18-, 36- and 54-month follow-up will abbreviated as P-18, P-36 and P-54 respectively. Similar convention is used for stool frequency (SF), tenesmus (T), and rectal bleeding (RB).

**Table 1:** **GI endpoints, the scoring system, baseline criteria for exclusion and the prevalence at 18-, 36- and 54-month follow-up**

**Scoring system selected endpoint**	**Baseline criteria & grade cut–point**	**Prevalence at**		
		**18-month**	**36-month**	**54-month**
**CTC v2.0** [21]				
Proctitis	>0	134/713	116/639	83/482
**LENT–SOMA** [22]				
Stool frequency	>0	264/716	220/639	168/487
Tenesmus	>0	198/716	174/638	116/487
Rectal bleeding	>0	134/716	140/639	114/487

CTC v2.0, Common Toxicity Criteria (version 2); LENT-SOMA, Late Effects of Normal Tissues-Subjective, Objective, Management, Analytic.

### Statistical analysis

The impact of treatment planning and delivery factors on the occurrence of GI toxicity was analysed by uni- and multivariate analysis. Univariate analysis was performed for endpoints to determine odds-ratio (OR). Unselected multivariate logistic regression analysis (with all factors included regardless of statistical significance in univariate analysis) was used to look for the relative contributions of the factors. Adjustments for potential confounders were made for age, risk category, body mass index (BMI) and the year of treatment. Because many variables were included in the analysis, the Benjamini & Hochberg false discovery rate (FDR) adjustment for multiple testing was performed [25] which is less conservative than the Bonferroni method. Specifically, we apply the Benjamini & Hochberg adjustment in R to map each *p*-value to a FDR-corrected *p*-value, which can be interpreted as the probability that the given factor is a false discovery [26]. FDR-adjusted *p*-values < 0.05 were considered statistically significant.

### DVH analysis

Each patient’s anorectum was delineated prior to data submission and archive, and manually reviewed and re-defined by SR and MK according to the ‘anorectum’ definition from Peeters *et al.* (defined as outer rectal wall from the level of the ischial tuberosities until when the rectum turns horizontally to the sigmoid colon) from which the inferior 3 cm were defined separately as the ‘anal canal’ and the remaining as the ‘rectum’ [27]. Doses for individual treatment phases were combined as equivalent dose in EQD2_3_ = 2 Gy [28], and an independent calculation undertaken in SWAN [19] of the anorectum relative cumulative dose-volume histogram (DVH). Dose/volume threshold indices (labelled as V1, V2 …) represent the relative proportions of anorectum/anal canal/rectum volume receiving more than a given dose in 1 Gy intervals. Median anorectum/anal canal/rectum DVH curves were derived for patient groups defined according to the significant variable categories or a dichotomisation of continuous variables about their median. The 95% confidence interval of the respective median volume at each EQD2_3_ point was derived from 10^4^ bootstrap samples. These DVH curves are not intended to be studied as one of the variables but rather to detect whether there are differences in dose distribution as a result of different treatment planning and delivery factors used. Comprehensive DVH analysis has been published elsewhere [29].

## Results

### Data and toxicity description

Distributions of investigated parameters are shown in Table 2. CI (phase 2), 95% isodose volume, and rectal volume were excluded from analysis because of the strong correlation (Spearman R > 0.8) to CI (phase 1), PTV and mean rectal CSA respectively. Table 2 shows the distribution of patients with and without toxicities for each endpoints at timepoints.

**Table 2:** **Distributions of investigated factors**

**Participants**	
Age	69 ± 7(49–85) years
BMI	27.98 ± 4.12(17.17-45.77) kg/m^2^
Year commenced EBRT	2004(125); 2005(200); 2006(234); 2007(181); 2008(12)
Risk category	Intermediate (464); High (290)
**Planning & Delivery factors**	
Dose calculation algorithm [2]	Type-*a* (401); Type-*b* (350)
Patient orientation at set-up	Prone (66); Supine (687)
Prescription dose	66Gy (99); 70Gy (423); 74Gy (229)
Treatment beam definition	Block/collimator (259); MLC (484)
Beam energy	6MV (96); 10MV (158); 15MV(5); 18MV (493)
Number of beams	3 (89); 4 (396); 5 (155); 6 (110);7 (2)
Rectal dietary intervention	No intervention (280); laxatives (77); bulking agent (394)
**Anatomical/Dosimetric parameters**	
PTV1 volume	192 ± 65 (30–704) cm^3^
95% isodose volume	257 ± 104 (60–1425) cm^3^
Conformity index [18]	1.34 ± 0.26 (0.72 – 2.94)
Rectal cranio-caudal length	9.6 ± 1.3 (5.4 – 13.5) cm
Mean rectal CSA	7.9 ± 3.4 (0.1 – 26.7) cm^2^
PTV-rectum separation	0.12 ± 0.32 (−2.6 – 3.39) cm

Continuous factors are specified as mean ± standard deviation (range), categorical factors are specified as category (number of patients). Not all patients available for all assessments due to missing data, exclusions etc. *Abbreviations*: *BMI* Body mass index, *EBRT* External beam radiotherapy, *PTV1* Phase 1 planning target volume (PTV), *CSA* Cross sectional area.

### Outcomes analysis

The results of univariate logistic analysis of treatment planning and delivery factors affecting late effects, with significant factors highlighted, are shown in Additional file 1. Some factors, including DCA, number of beams and beam energy found to be significantly associated with at least 3 endpoints but none of the factors remain significant after FDR adjustments. The multivariate results for significant factors are in Table 3. At 36-month follow-up post-randomisation, patients planned with type-*b* DCA were associated with lower risk of having increased SF-36 (OR: 0.435, CI: 0.242-0.783, FDR-adjusted *p* = 0.04). At 54-month, a similar association was found albeit weaker (unadjusted *p*-value < 0.10). Similar trends towards significance were also found between the DCA and RB-36 and RB-54). Patients using laxatives as a pre-treatment rectal preparation had higher risk of SF-36 compared to patients with no rectal dietary intervention (OR: 3.639, CI: 1.505-8.818, FDR-adjusted *p* = 0.04). The effect of laxative as a pre-treatment rectal preparation was also found to show a trend toward significance for SF-18 and T-18. Planning target volume (PTV), conformity index, rectal width and prescription dose were not significant when adjusted for false discovery rate. However, special attention is given to PTV. Before adjustment for multiple comparisons [25], increasing PTV is significantly associated with increasing risk of SF at 18- and 54-month while at 36-month the association shows a suggestive trend (*p* = 0.06). Number of beams, beam energy, treatment beam definition, positioning orientation, rectum-PTV separation, rectal length and mean cross sectional area did not affect the risk of toxicities in multivariate analysis.

**Table 3:** **Multivariate analysis examining relationship between treatment factors to late gastrointestinal toxicities**

	**18 months**				**36 months**				**54 months**			
**Endpoint**	**Variable**	**P**	P*	**OR (CI)**	**Variable**	**P**	P*	**OR (CI)**	**Variable**	**P**	P*	**OR (CI)**
**Proctitis**	Orientation (prone vs supine)	0.051	NS	2.467 (0.996–6.111)	Conformity index (continuous)	0.032	NS	3.157 (1.102–9.046)				
	Mean CSA (per cm^2^)	0.062	NS	0.926 (0.854–1.004)								
**Rectal bleeding**					Dose calculation algorithm (type-*b* vs type-*a)*	0.011	NS	0.479 (0.271–0.847)	Dose calculation algorithm (type-*b* vs type-*a*)	0.055	NS	0.561 (0.311–1.013)
					Rectal width (per cm)	0.026	NS	0.726 (0.548–0.963)				
					Prescription dose (per Gy)	0.021	NS	0.876 (1.017–1.158)				
					PTV (per cm^3^)	0.061	NS	1.003 (1–1.007)				
**Stool frequency**	Rectal intervention (bulking agent vs no intervention)	0.049	NS	0.78 (0.436–1.395)	Dose calculation algorithm (type-*b* vs type-*a*)	0.006	0.039	0.435 (0.242–0.783)	Dose calculation algorithm (type-*b* vs type-*a*)	0.079	NS	0.554 (0.287– 1.07)
	Rectal intervention (laxative vs no intervention)	0.053	NS	1.749 (0.801–3.819)	Rectal intervention (laxative vs no intervention)	0.003	0.039	3.639 (1.502–8.818)	Number of beam (per 1 beam)	0.083	NS	0.692 (0.457–1.049)
	PTV (per cm^3^)	0.022	NS	1.004 (1.001–1.007)	PTV (per cm^3^)	0.059	NS	1.004 (1–1.007)	PTV (per cm^3^)	0.008	NS	1.007 (1.002–1.012)
	PTV–rectal separation (per cm)	0.061	NS	1.84 (0.971–3.486)	Conformity index (continuous)	0.049	NS	0.293 (0.087–0.993)				
**Tenesmus**	Number of beam (per 1 beam)	0.085	NS	0.775 (0.581–1.036)								
	Rectal intervention (laxative vs no intervention)	0.072	NS	2.058 (0.971–4.364)								

*Abbreviations*: *P* *p*-value, P* False-discovery-rate-adjusted *p*-value [25], *OR (CI)* odds ratio (95% confidence interval), *CSA* Cross sectional area, *PTV* Planning target volume, *NS* not significant. Only variables with uncorrected *p*-value <0.10 are shown.

### DVH comparisons

**Figure 1:**
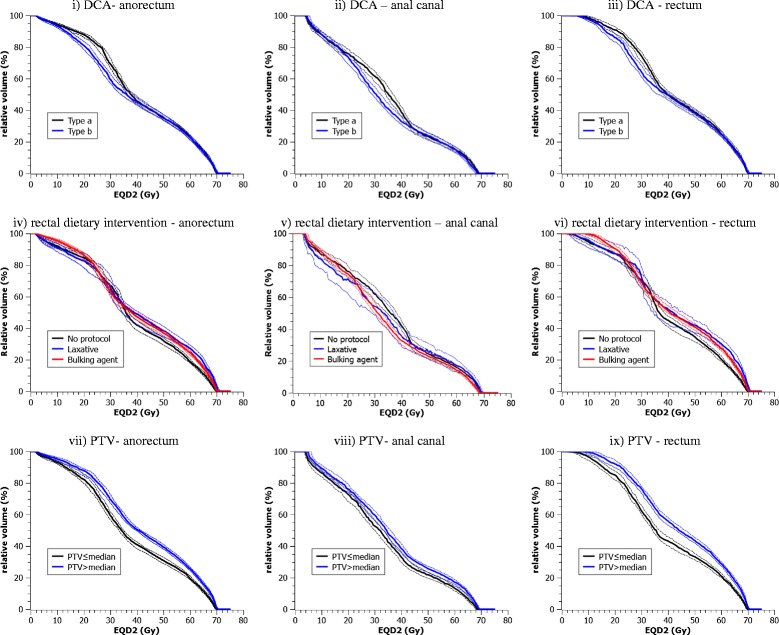
**Median anorectum, anal canal and rectum dose volume histogram (DVH) curves according to the dose calculation algorithm (DCA), rectal dietary intervention and PTV.** The dashed lines are the 95% confidence interval of the respective median derived from 10^4^ bootstrap samples.

Median anorectum, anal canal and rectum DVH curves for all patients in the study according to the treatment factors of interests are shown on Figure 1 (i-ix). At lower doses, it was found that the difference of the median relative volume of the anorectum receiving between EQD2_3_ = 15 Gy and EQD2_3_ = 35 Gy is significant between plans using type-*a* and type-*b* with maximum difference of 8% at V25; lower for type-*b*. However, at higher doses the differences were unremarkable. The same is also seen for anal canal and rectum DVHs. For dietary intervention, median DVHs were found to be unremarkably different for patients administered with pre-simulation treatment laxative compared to those with no dietary intervention or prepared with a bulking agent. Consistently higher median volumes were found for patients with PTV ≤ median compared to > median especially for anorectum and rectum DVHs.

## Discussion

In the current study, we report on the treatment planning, delivery and anatomical factors impacting late GI toxicities following external beam RT of the prostate.

This study has several unique and interesting features including the large number of patients with full data collation and the diversity of treatment technique across 23 institutions. Data was prospectively collected in a clinical trial setting, monitored by strict quality control and auditing requirements [16]. As the study was performed in the era of transition to fully 3-dimensional conformal radiotherapy (3DCRT), some modifications (e.g. DCA and beam-shaping devices) were captured as centres transitioned into newer technology. This unique opportunity allows the variations to be captured in a single study enabling analysis on a variety of treatment factors in the multivariate analysis setting. Additionally, toxicity-specific late endpoints were analysed to allow a more comprehensive analysis. This is unlike many other reports which focus on a single specific GI toxicity (e.g. rectal bleeding [6,30]) or use non-atomised symptoms grades (e.g. Radiation Therapy Oncology Group [RTOG] scale [3,4,31]).

We found that in multivariate analysis, several factors were found to affect the toxicity outcome.

1. Higher odds of stool frequency was found for patients administered with laxative compared to patients with no rectal preparatory intervention at 36-month follow-up and potentially at 18-month. Patients administered with laxative had unremarkable difference of planned dose distribution compared to patients who did not receive any interventions indicating that the decreased toxicity risk did not originate from differences in the planned dose distribution.
  There are potentially two ways to look at this observation. First, compared to patients with no rectal intervention, laxative use helps to minimize the systematic error due to rectum motion during pelvic radiotherapy [32,33] but this finding is not repeated in a randomised study [5]. This careful and more thorough emptying of rectum by laxative may also spare the rectal tissue from the benefit of the blurring effect due to the day-to-day spatial variations. Second, the intervention may have drug-radiation synergistic effect to increase damage to the intestinal villi caused by radiotherapy. Lips *et. al* found a trend towards slightly more gastrointestinal toxicity in the laxative arm than in the placebo arm in a randomised study [5].
2. Association of type-*a* DCA used in planning to increased risks of stool frequency may be related to the dose distribution difference between the two DCAs. The disagreements between type-*a* and type-*b* has been proposed to be insignificant in prostate radiotherapy treatment because tissues in the pelvic region are generally homogeneous [34]. However, a treatment planning study comparing the effect of algorithm on the normal tissue complication probability (NTCP) model illustrated that the difference was not insignificant, with the use of type-*b* resulting in lower NTCP when the same monitor units were used [35].
3. We found that patients with smaller PTV had less risk of gastrointestinal toxicity. This is similar to previous observations [9,30,31] and is potentially attributable to the more favourable dose distribution for patients with smaller PTV.

Several factors found to be significant in previous studies were not found to be significant in this study, including rectum CSA [6,30] and rectal length [6] potentially because of the difference in endpoint definition used. Prevalence, rather than cumulative incidence, at three late time points were used in this study to better reflect the actual toxicity burden and to reduce noise [23,24]. From the multivariate analysis shown here, it was found that several factors showed low *p*-values (*p* < 0.05, uncorrected) at a specific time point but were not repeated at other time points raising the suspicion that it might be due to data fluctuation or random discovery rather than actual long term associations. The confidence to report the associations between dietary intervention and DCA to toxicity risks, however, was greater as these factors were also found to have trend towards significance in other time points. This suggests sustained long-term associations.

The RADAR trial provided a rich dataset in terms of the variety of patient treatment and irradiation techniques used, resulting in diverse combinations of treatment approaches and dose distributions. All treatments were however based on 3DCRT and it is important to consider how the results derived here may be translated to patient treatment with image-guided and intensity-modulated radiotherapy (IMRT), which are now commonplace. IMRT produces a more conformal dose distribution to 3DCRT, enabling higher doses to be prescribed in larger dose per fraction while maintaining or decreasing the associated toxicities [7]. Firstly, the rectal preparatory interventions discussed in this study are still widely used in a more contemporary treatment delivery including IMRT [36]. We have speculated that the elevated risk of toxicity related to the use of laxative may have an origin largely unrelated to dosimetry. It is thus expected that the result is applicable to treatments using IMRT. A comparable effect of using laxative was also reported in a randomised study with IMRT as a method of treatment delivery using 77 Gy and 2.2 Gy/fraction regimen [5]. Secondly, the use of many small beam elements in IMRT to create steep and conforming dose gradients may profit more from the use of a more rigorous DCA [37]. While DCA has not been previously reported to affect homogenous tissue such as found in the pelvis [34], conflicting NTCP results have been observed [35]. Finally, better dose sculpting associated with IMRT delivery may reduce the impact of the PTV on stool frequency compared to what is observed in this study. Stool frequency is more strongly associated with dose to the anal canal [29]. Due to the distance of the anal canal from the PTV, relevant doses could be reduced by employing a treatment technique with sharper dose fall-off. This may in part be compensated by the use of higher dose prescriptions, often aimed at maintaining iso-effect in normal tissues. It is unknown what the potential impacts might be of hypofractionation, common in modern treatments, on the relationships found here. As the α/β estimate for rectal toxicity is likely higher than that for prostate cancers, leading to a constant or lower proportional rectal toxicity rate in hypofractionation [38], it can be speculated that a less significant relationship between the treatment factors considered here and toxicity might be observed.

As an alternative to undertaking a randomised controlled trial of a specific technical modification, this study has demonstrated the benefit of careful archival of treatment demographics to reveal the clinical impact of treatment modifications. Even when datasets from clinical trials are not originally powered to assess the effects of these incremental technical modifications, such assessment may offer some valuable clinical insights [10]. With technical modifications typically introduced into practice incrementally and uncritically, derivation of Level III randomised trial evidence for the impact of such modifications is unlikely [39]. Consequently, little or no evidence is available regarding the existence or size of clinical impact of some of the technological modifications studied here. In most cases, the modifications were introduced with the immediate aim of improving treatment quality on the basis of prior successful planning dosimetric studies [1].

The notion to supplement the evidence from dosimetric studies with secondary analysis of the data from clinical trials to determine the clinical consequence is not new. Clinical trials data is associated with rigorous scrutiny, consistent adherence to protocol, quality assurance procedures and potentially less noise compared to retrospective analysis of day-to-day clinical cases making it an appropriate dataset for that purpose [10,16,39]. For example, the evidence of the benefit of the transition from 3DCRT to IMRT and image-guided radiotherapy has been successfully complemented by secondary analyses of clinical trials [7,8]. With more trial registries being encouraged to record the technical modifications introduced, the influence of technical treatment planning and delivery factors that may be clinically relevant can be studied more confidently [39].

The insights obtained in this study are hypothesis-generating and require independent validation. Specific limitations in our analysis should be highlighted. The factors are not independent of each other, with many factors grouping together due to the technique, experience and manufacturer-dominated profiles of participating centres. It should also be noted that the influencing factor of centre reporting bias cannot be excluded.

## Conclusion

The RADAR study dataset has allowed an assessment of treatment and anatomical factors previously hypothesised to impact on treatment-related GI toxicity. A limited number of significant associations were subsequently found which cannot always be explained by the underlying planned dosimetry. The ability to undertake this analysis was facilitated by careful collection of treatment and demographic data during the undertaking of the trial. Consequently, trial registries should be encouraged to record the technical modifications introduced.

## Additional file

Additional file 1:
**Univariate analysis examining relationship between treatment factors to late gastrointestinal toxicities at 18-, 36- and 54-month follow-up.**

## Abbreviations

RADAR: Randomised androgen deprivation and radiotherapy
DCA: Dose calculation algorithm
OR: Odds ratio
DVH: Dose-volume histogram
EQD2_3_: Equivalent dose in 2Gy per fraction using α/β = 3
PTV: Planning target volume
RT: Radiotherapy
GI: Gastrointestinal
QA: Quality assurance
CT: Computed tomography
CTV: Clinical target volume
PTV1: Planning target volume in phase 1
PTV2: Planning target volume in phase 2
CI: Conformity index
MLC: Multileaf collimator
CSA: Cross sectional area
FDR: False discovery rate
3DCRT: 3-Dimensional conformal radiotherapy
RTOG: Radiation Therapy Oncology Group
NTCP: Normal tissue complication probability
