# Supplementary material for: Impact of treatment planning and delivery factors on gastrointestinal toxicity: an analysis of data from the RADAR prostate radiotherapy trial
Source: Radiat Oncol. 2014 Dec 13;9:282. doi: 10.1186/s13014-014-0282-7 (PMC4271488; doi:10.1186/s13014-014-0282-7)
Supplement: Additional file 1: — Univariate analysis examining relationship between treatment factors to late gastrointestinal toxicities at 18-, 36- and 54-month follow-up. [file 13014_2014_282_MOESM1_ESM.doc]

Additional file 1: Univariate analysis examining relationship between treatment factors to late gastrointestinal toxicities at 18-, 36- and 54-month follow-up

|  | **Proctitis** | | | **Rectal bleeding** | | | **Stool frequency** | | | **Tenesmus** | | |
| --- | --- | --- | --- | --- | --- | --- | --- | --- | --- | --- | --- | --- |
|  | **18** | **36** | **54** | **18** | **36** | **54** | **18** | **36** | **54** | **18** | **36** | **54** |
| **Factors** | **Odds ratio (OR)** | | | | | | | | | | | |
| **Prescription dose (/1 Gy)** | 1.01 | 1.02 | 0.96 | 1.01 | 0.94 | 0.97 | 0.96 | 0.97 | 0.97 | 1.00 | 0.99 | 0.99 |
| **Orientation (prone vs supine)** | 1.49 | 1.43 | 1.70 | 1.08 | 1.03 | 1.15 | 1.47 | 0.79 | 0.60 | ***0.42*** | 0.66 | 0.91 |
| **Conformity index (/1 unit)** | 0.91 | ***2.31*** | 1.76 | 0.82 | 1.45 | 0.89 | 0.83 | 0.64 | 0.69 | 0.61 | 0.96 | 0.78 |
| **Beam energy (/1 MV)** | 1.02 | 0.97 | 1.00 | 1.03 | 1.02 | 1.04 | 1.01 | 1.03 | ***1.06*** | ***1.07*** | 1.04 | ***1.08*** |
| **PTV-rectum separation (/1 cm)** | 1.15 | 0.60 | 0.87 | 1.41 | 0.88 | 0.62 | 1.36 | 0.83 | 1.09 | 1.22 | 0.92 | 1.42 |
| **Rectal distension (/1 cm)** | 0.94 | 0.95 | 0.84 | 0.84 | ***0.79*** | 0.99 | 0.89 | 1.00 | 0.97 | 0.89 | 1.06 | 0.92 |
| **Mean rectal cross sectional area (/1 cm^2^)** | 0.94 | 0.99 | 0.99 | 0.94 | 0.98 | 1.04 | 0.98 | 0.98 | ***0.93*** | ***0.93*** | 0.99 | 0.94 |
| **Dose calculation algorithm (type-*b* vs type-*a*)** | 0.88 | 1.00 | 0.68 | 0.84 | ***0.72*** | 0.56 | 0.72 | ***0.61*** | ***0.54*** | 1.11 | 0.83 | 0.82 |
| **Laxative vs no intervention** | 0.90 | 0.74 | 0.89 | 1.39 | 0.73 | 0.96 | 1.01 | 1.65 | 0.98 | ***2.75*** | 1.53 | 2.01 |
| **Bulking agent vs no intervention** | 1.14 | 0.96 | 0.85 | 1.61 | 1.07 | 0.98 | ***0.63*** | 0.84 | 0.75 | 1.78 | 1.39 | 2.12 |
| **PTV1 (/1 cm^3^)** | 1.00 | 1.00 | 1.00 | 1.00 | 1.00 | 1.00 | 1.00 | 1.00 | 1.00 | 1.00 | 1.00 | 1.00 |
| **Number of beams (/1 beam )** | 0.84 | 0.98 | 0.88 | 0.91 | 0.86 | ***0.74*** | 0.92 | 0.80 | ***0.65*** | ***0.76*** | ***0.77*** | ***0.71*** |
| **Rectal length (/1 cm)** | 1.05 | 0.89 | 1.13 | 1.06 | 0.98 | 0.94 | 0.95 | 0.95 | 0.98 | 1.07 | 0.95 | 1.11 |
| **Treatment beam definition (MLC vs block)** | 1.17 | 1.08 | 1.23 | ***1.83*** | 1.05 | 1.11 | 0.71 | 0.62 | 0.74 | 1.05 | 1.23 | 1.24 |

Factors with uncorrected *p*< 0.05 were bold. None of the factors have adjusted *p*< 0.05. Abbreviations: CSA- cross sectional area; PTV- planning target volume.
